# Putting the Brakes on Huntington Disease in a Mouse Experimental Model

**DOI:** 10.1371/journal.pgen.1005409

**Published:** 2015-08-06

**Authors:** Jane C. Kim, Sergei M. Mirkin

**Affiliations:** Department of Biology, Tufts University, Medford, Massachusetts, United States of America; St Jude Children's Research Hospital, UNITED STATES

**Fig 1:**
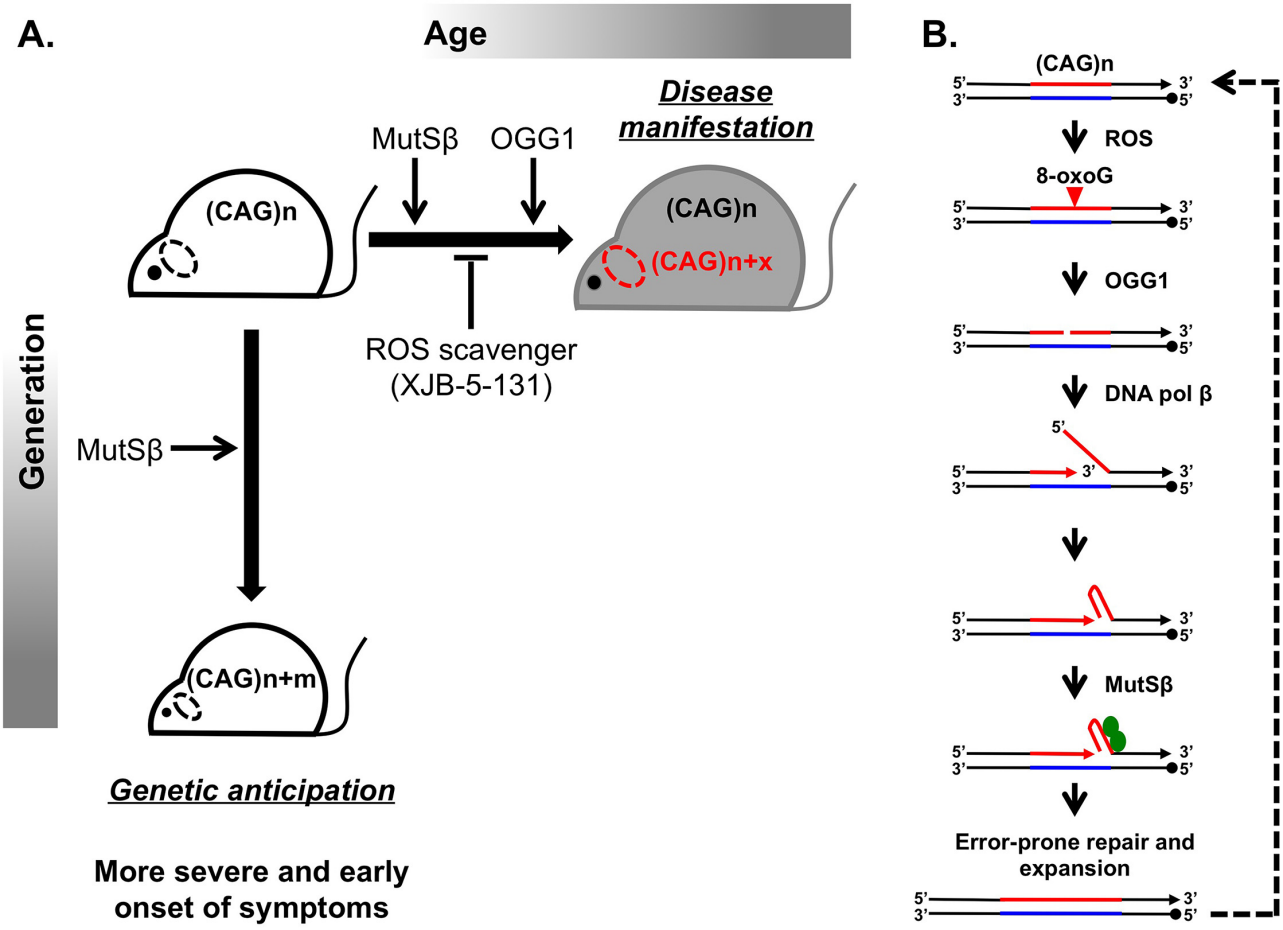
Inhibiting somatic expansions delays onset of disease in a Huntington mouse model. (A) Expanded (CAG)_n_ repeats are more susceptible to undergoing further expansions, contributing to two distinct aspects of their instability. Intergenerational transmission (vertical axis) is the general increase in repeat length (m) from parent to offspring, which has been found to be dependent on MutSβ in mice. Somatic expansions (horizontal axis) are an increase in repeat length beyond the inherited length throughout the lifetime of an individual (x) and are dependent on both MutSβ and 8-oxoguanine glycosylase (OGG1) in mice. Somatic expansions exhibit tissue-specific differences and, in HD, occur predominantly in the brain (dotted oval). The absence of OGG1 significantly delays the age of disease onset in Hdh mice. This delayed onset of disease symptoms is recapitulated by inhibiting somatic expansion with a ROS scavenger, XJB-5-131. (B) Aberrant repair of DNA oxidative damage at (CAG)_n_ repeats promotes somatic expansions. Initiation of 8-oxoguanine (8-oxoG) base excision repair by OGG1 leads to a nick in the damaged DNA strand. Subsequent strand-displacement synthesis by DNA polymerase β creates a 5ʹ-flap. A hairpin formed by CAG repeats in this flap could be stabilized by MutSβ and incorporated into the repaired DNA to generate an expansion. This expanded region is further subjected to ROS, possibly generating “toxic oxidation cycles” (dotted line).

Huntington disease (HD) is a hereditary neurodegenerative disorder that causes a progressively debilitating impact on movement, cognition, speech, and mood. It most commonly develops during adulthood and worsens over a 10–15-year period. The genetic basis of HD is an expansion of the (CAG)_n_ trinucleotide repeat in the first exon of the *HTT* gene [1,2]. Although the function of the normal HTT protein is not well established, in-frame repeat expansion results in the accumulation of an abnormally long polyglutamine tract, which is believed to contribute to mutant protein toxicity and neural degeneration [3]. Consequently, CAG repeat length is inversely correlated with age of onset and severity of disease. Disease-size CAG repeats are prone to further lengthening, which leads to two distinct aspects of their instability: expansions during intergenerational transmissions and somatic expansions occurring throughout the lifetime of an individual (Fig 1A).

An outstanding question in the field is whether somatic expansions contribute to disease. The problem is that somatic expansions occur in the context of expressing an already toxic protein, making it difficult to address this question. Two commonly discussed ideas are that (i) disease arises with time because of expression of the toxic protein or RNA or that (ii) the onset of disease depends on gradual accumulation of somatic expansions in patients’ tissues throughout life. To distinguish between these scenarios, it is critical to assess to what extent, if any, somatic expansions accelerate disease progression.

Since 1993 when the cause of HD was first reported, substantial effort has been made to understand the molecular basis of repeat expansions and the mechanisms of disease pathophysiology. While these studies were conducted in various model systems [4,5], mouse models appeared to be particularly well suited for unraveling disease pathophysiology. Despite a few caveats, such as the much longer CAG repeat lengths (i.e., >100 repeats) needed to recapitulate disease symptoms and the relatively small-scale of expansions, mouse models of HD have led to principal contributions in the field. First, they exhibit both intergenerational and somatic repeat expansions. Second, candidate gene analysis uncovered a surprising role of the mismatch repair (MMR) complex MutSβ in promoting rather than protecting against CAG repeat expansions (Fig 1) [6,7].

A breakthrough in distinguishing between the molecular mechanisms of intergenerational and somatic expansions came with the discovery that loss of 8-oxoguanine glycosylase (OGG1) specifically decreased CAG expansions in somatic cells, but it had no effect on intergenerational transmissions [8]. The main function of OGG1 is to remove 8-oxoguanine (8-oxoG), a mutagenic base by-product accumulating in DNA after its exposure to reactive oxygen species (ROS). It was hypothesized, therefore, that aberrant repair of DNA oxidative damage specifically elevates repeat instability in long-lived differentiated cells, such as neurons. OGG1 excises 8-oxoG, generating a nick in the damaged DNA strand, which is further processed to permit DNA repair synthesis. DNA polymerase β then conducts strand-displacement synthesis, creating a 5′-flap. Because CAG repeats are prone to hairpin formation, a hairpin formed in the flap could be stabilized by the MutSβ complex and incorporated into the repaired DNA to generate an expansion. This expanded region is further subjected to ROS, possibly generating “toxic oxidation cycles” [8] in which many repeats can be added over time (Fig 1B). In nondividing cells, repetitive hairpins can only be formed during DNA repair synthesis; hence, repeat expansions are triggered by their oxidative damage followed by aberrant base excision repair. In dividing cells, repetitive hairpins are readily formed during DNA replication [9], which makes expansions during intergenerational transmissions independent of OGG1.

The work of Budworth et al. in this issue of *PLOS Genetics* addresses the role of somatic expansions in disease pathophysiology by further characterizing OGG1 null mice [10]. In effect, this knockout serves as a convenient separation of function tool, since it affects somatic but not intergenerational expansions of CAG repeats in the HD mouse. This is an important advance from previous studies knocking out MMR proteins in mice because it is difficult to distinguish the contribution from both processes on disease progression. They found that decreasing somatic expansion in OGG1-deficient mice delays the onset of disease symptoms, as assessed by endurance tests such as rotorod performance and grip strength analysis, compared to OGG1^+^ littermates that inherit the same disease-length allele. This conclusion is hard won, since there are several challenges to addressing somatic instability and disease progression. Primarily, there is large variability in both expansion sizes and behavioral outcomes among individual mice. Furthermore, the observed changes in the average repeat lengths are relatively small. The authors deal with these challenges through their experimental design and data analysis. First, they evaluate a large sample size to increase statistical power (1,200 animals). Second, they bin age groups of 5 or 10 weeks and assess behavioral performance before obtaining tissue to test repeat length. This strategy seems superior to tracking a group of animals over time, as it precludes the influence of sampling bias on testing outcomes. Finally, the authors employ statistical tests that look for differences between two repeat length distributions rather than their average values alone.

Because decreasing somatic expansions in OGG1 knockouts delayed disease progression, Budworth et al. attempted to pharmacologically inhibit somatic expansion by treating HD mice with a mitochondrial scavenger of ROS called XJB-5-131. As previously reported, this treatment improved rotarod performance in Hdh (Q150/Q150) mice [11]. Here, the authors demonstrate that these mice also show decreased somatic expansions (Fig 1A). Taken together, these results are strongly indicative that somatic expansions contribute to HD progression, specifically to the “when” rather than “if” of disease onset. As a word of caution, a formal possibility still remains that the absence of OGG1 or decreased ROS could affect HTT toxicity indirectly. These findings are very promising in opening up a new therapeutic target area, since XJB-5-131 or similar pharmacological inhibitors of oxidative damage should have minimal toxic effects and little increase in genomic instability.

HD is just one example of more than 30 diseases caused by unstable microsatellite sequences, many of which also demonstrate somatic instability. It is unclear how the mechanism of repeat instability differs for different repetitive sequences or even for the same repeat located at other genomic loci. Testing OGG1-dependent somatic instability in mouse models of different repeat expansion diseases such as myotonic dystrophy caused by (CTG)_n_ repeats, fragile X syndrome caused by (CGG)_n_ repeats, spinocerebellar ataxia type 10 caused by (AATCT)_n_ repeats, and several spinocerebellar ataxias caused by other (CAG)_n_ repeats will shed light on the extent to which inhibiting oxidative damage may be a common therapeutic course for different repeat expansion diseases. As a first pass, XJB-5-131 treatment will be a useful discriminator to implicate oxidative damage in somatic expansion of different microsatellite sequence. It remains to be seen what is the contribution of various molecular mechanisms on overall somatic instability in HD, but the work from Budworth et al. provides the important foundation that inhibiting somatic expansion is a worthwhile intervention and endeavor toward improving HD patient care.
